# Calf Diarrhea Is Associated With a Shift From Obligated to Facultative Anaerobes and Expansion of Lactate-Producing Bacteria

**DOI:** 10.3389/fvets.2022.846383

**Published:** 2022-03-22

**Authors:** Diego E. Gomez, Lynna Li, Hanne Goetz, Jennifer MacNicol, Lisa Gamsjaeger, David L. Renaud

**Affiliations:** ^1^Department of Clinical Studies, Ontario Veterinary College, University of Guelph, Guelph, ON, Canada; ^2^Department of Population Medicine, Ontario Veterinary College, University of Guelph, Guelph, ON, Canada; ^3^Department of Animal Sciences, Ontario Agricultural College, University of Guelph, Guelph, ON, Canada; ^4^Vetsuisse Faculty, Department for Farm Animals, Division of Ruminant Medicine, Zurich, Switzerland

**Keywords:** dysbiosis, D-lactate, L-lactate, anion gap acidosis, acidemia

## Abstract

Diarrhea is the leading cause of morbidity, mortality and antimicrobial drug use in calves during the first month of age. Alteration in the bacterial communities of the gastrointestinal tract occurs during diarrhea. Diarrheic calves often develop anion gap (AG) acidosis associated with increased concentrations of unmeasured anions including D- and L-lactate. However, studies investigating the association between gut microbiota alterations and the development of acid-base disorders in diarrheic calves are lacking. We investigated the fecal bacterial alterations of calves with diarrhea and its association with changes in blood pH, and AG. Blood and fecal samples from healthy and diarrheic veal calves were taken 7 days after arrival to the farm. The fecal microbiota of healthy and diarrheic calves was assessed by sequencing of 16S ribosomal RNA gene amplicons. Blood gas analysis was completed using an i-Stat analyzer. In healthy calves, higher richness, evenness, and diversity were observed compared to diarrheic calves. *Phocaeicola, Bacteroides, Prevotella, Faecalibacterium, Butyricicoccus*, Ruminococcaceae and Lachnospiraceae were enriched in healthy compared with diarrheic calves. *Enterococcus, Ligilactobacillus, Lactobacilus, Gallibacterium Streptococcus*, and *Escherichia/Shigella* were enriched in diarrheic calves. In diarrheic calves, an increased abundance of lactate-producing bacteria including *Lactobacillus, Streptococcus, Veillonella, Ligilactobacillus* and *Olsenella* was detected. Diarrheic calves had a lower pH and bicarbonate concentration and a higher AG concentration than healthy calves. Together, these results indicate that calf diarrhea is associated with a shift from obligated to facultative anaerobes and expansion of lactate-producing bacteria which are related to acidemia, low bicarbonate and increase AG. Our results highlight the importance of the gastrointestinal microbiota on the clinicopathological changes observed in diarrheic calves.

## Introduction

The U.S. National Animal Health Monitoring System reported that 32% of calf mortality in 2018 was caused by diarrhea during the first 3 weeks of life (1). Calf diarrhea is predominantly caused by infectious agents, such as viruses, bacteria, and protozoa. The main causative agents of calf diarrhea include bovine rotavirus group A, bovine coronavirus, *Salmonella* spp., Enterotoxigenic *Escherichia coli* (ETEC), *Clostridium perfringens* type C, and *Cryptosporidium parvum* (2). Regardless of the cause of diarrhea, reduced bacterial diversity and changes in the gut bacterial populations from normal have reported in diarrheic calves (3). In calves, diarrhea is associated with an increase in taxa from the phylum Proteobacteria, especially increased abundance of Enterobacteria (4–6) and decreased abundance of butyrate-producing bacteria (e.g., *Bifidobacterium* and *Faecalobacterium*) (3, 7). In humans, a reduction in butyrate producing obligate anaerobes such as *Faecalibacterium prausnitzii* (8–10) and an increase in facultative anaerobes such as *Escherichia coli* appears to be a feature of gastrointestinal diseases (8–10). It has been hypothesized that this shift from obligate anaerobes to facultative anaerobes results from an increased release of hemoglobin carrying oxygen and reactive oxygen species into the lumen of the gastrointestinal tract (oxygen hypothesis), which favor the growth of facultative anaerobes (11). Therefore, the first objective of this study was to investigate the fecal bacterial alterations of calves with acute diarrhea (<48 h (h) duration) to determine whether a shift from obligatory to facultative anaerobes occurs in diarrheic calves.

Metabolic acidosis (decreased blood pH and HCO${}_{3}^{-}$ concentration) is the most common acid-base disorder occurring in calves with diarrhea (12). Metabolic acidosis is usually accompanied by an increased anion gap (AG), which represents the presence of unmeasured anions (UA) in plasma, commonly D- and L-lactate (6, 7, 12–14). The mechanisms resulting in increased concentrations of UA in diarrheic calves are not fully understood; however, the concentration of D- and L-lactate in fecal samples of diarrheic calves is higher than that of healthy calves (13). These findings suggest that gut bacterial fermentation could contribute to the development of AG acidosis in diarrheic calves. Therefore, the second objective of this study was to investigate if there is a relationship between the fecal microbial alterations of diarrheic calves and the changes in blood pH, bicarbonate, and the load of unmeasured ions calculated using the Anion Gap (AG).

## Materials and Methods

### Calf Housing and Feeding

This study was conducted at a commercial grain-fed veal producer in southern Ontario, Canada. The farm was selected based on its proximity to the University of Guelph and willingness to participate in the study. Calves arrived at the facility in 2 batches of 80 Holstein calves which arrived on December 21, 2020, and January 11, 2021. The age of the calves ranged from 3–10 days of age. They were sourced from local dairy farms, auctions, and a drover, and, after arrival to the facility, were housed in individual pens. Calves were offered milk replacer (26% protein and 20% fat with 2.4% lysine and 0.8% methionine whey protein concentrated based milk replacer without any feed additives) twice daily using the following schedule: week 1: 650 g mixed in 5 L of water/day; week 2: 780 g in 6 L/day; week 3: 910 g in 7 L/day; week 4 and 5: 1040 g in 8 L/day; week 6: 780 g in 6 L/day; week 7: 520 g in 4 L/day; and week 8: 325 g in 2.5 L/day. Calves were also offered texturized calf starter [20% crude protein (CP)] upon arrival until week 8. Both the milk replacer and the starter were medicated with decoquinate.

### Data Collection and Outcomes

Upon arrival to the facility, calves were weighed using a digital scale. Body weight was also taken at 7 days following arrival. In addition, all antimicrobial and supportive treatments were recorded when administered to each calf. Calves were scored daily for fecal consistency by farm staff over the first 28 d after arrival using the following scoring method: fecal score 0 = normal consistency; 1 = semi-formed or pasty; 2 = loose feces; 3 = watery feces (14). Calves with a fecal score of 2 or 3 were classified as having diarrhea. At d 7 following arrival to the facility in both groups, researchers visited the farm to collect fecal and blood samples for blood gas and electrolyte analyses. Calves were classified as “healthy” if they did not have an abnormal fecal consistency score over the previous 7 d at the facility. Calves were classified as having diarrhea if they had a fecal score of 2 or 3. For the calves that fell under these specific classifications, fecal samples were collected fresh from calves upon rectal stimulation into Corning™ Falcon™ 15 mL Polystyrene Conical Centrifuge Tubes. The samples were transported on ice to the University of Guelph where they were stored at −20°C until analysis. Blood samples were collected from the jugular vein of the calf using a 20-gauge 1-inch needle into a 3 ml lithium heparin syringe (Vyaire Medical, Inc. Mettawa, IL, USA). The sample was immediately inserted into an EC8+ cartridge (Abbott, Mississauga, ON, Canada) and analyzed using an i-Stat analyser (Abbott, Mississauga, ON, Canada). From the analyser, the concentration of sodium (mmol/L), potassium (mmol/L), chloride (mmol/L), glucose (mmol/L), blood urea nitrogen (mmol/L), and haematocrit (%), pH, and venous pressure of carbon dioxide (PvCO_2_; mmHg) were determined and recorded. In addition, base excess (mmol/L) and the plasma concentration of hemoglobin (Hb, g/dL) and HCO_3_ (mmol/L) were calculated. The AG (mmo/L) was calculated using the formula: AG = (Na^+^ + K^+^) – (Cl^−^ + HCO${}_{3}^{-}$).

### Sample Size Calculation

A minimum sample size of 6 calves was established to detect a 25% change in operational taxonomic unit (OTU) counts, assuming a normal distribution with a mean ± SD OTU count of 2886 ± 391 per sample, a power of 0.80 and alpha of 0.05 (15).

### Sample Processing

The bacterial DNA from frozen fecal samples were extracted using the E.Z.N.A Stool DNA Kit (Omega Bio-Tek, Norcross, GA, USA) according to the manufacturer's protocol. The V4 region of the 16s rRNA gene was amplified via PCR, using modified 515-F and 806-R primers. Amplification was completed in a 25 μl reaction consisting of 12.5 μl of KAPA 2G Fast HotStart ReadyMix 2X (KapaBiosystems, MilliporeSigma, ON, Ca), 9.0 μl of molecular-grade water, 2.5 μl template DNA, and 0.5 μl each of both the forward and reverse primers (10.0 μM). The reaction conditions for PCR were 94 °C for 10 min, and 27 cycles of 94 °C for 45 s, 53 °C for 60 s, and 72 °C for 90 s, followed by a final period of 72 °C for 10 min. Using the Mag Bind RXNPure Plus Beads, the PCR products were then purified following the manufacturer instructions. The library pool was sequenced on an Illumina MiSeq for 250 cycles from each end conducted at Guelph's Agriculture and Food laboratory (Guelph, ON, Canada).

### Bioinformatic and Statistical Analysis

Bioinformatic analysis was conducted using the Mothur software (1.45.3) following a previously published protocol (16, 17). The Ribosomal Database Project classifier was used to identify and ensure that the sequences passed quality control. The sequences were then clustered at the genus level and clustered into Operational Taxonomic Units (OTUs). The value at which we sub-sampled was chosen based off the sample with the fewest reads and to ensure sub-sampling representation, Good's coverage for each sample was calculated (18). To evaluate the alpha diversity of the fecal microbiota, chao (richness), Shannon's evenness (evenness), and inverse Simpson (diversity) was calculated. The values were then compared using the non-parametric Steel-Dwass test for multiple comparisons. The Jaccard (19) and Yue & Clayton (20) indices were calculated to determine community membership and structure, respectively, of the fecal microbiota of healthy and diarrheic calves. Principal Coordinates Analysis (PCoA) plots were then generated using JMP 16.0 based on the Jaccard and Yue & Clayton indices to characterize clustering of different groups and the analysis of Molecular Variance (AMOVA) was used to determine differences between groups. Dirichlet multinomial mixtures method (DMM) was used to determine the number of metacommunities (distinct groups of samples with similar microbial composition) that the data could be clustered into (21). Relative abundance of the predominant taxa at the phylum, class, order, family, and genus levels were calculated and compared using the Steel-Dwass test for multiple comparisons and *P* values were adjusted using the Benjamini-Hochberg correction (22) (R! Core Team, 2013, R Foundation for Statistical Computing, Vienna, Austria). Linear discriminant analysis Effect Size (LEfSe) (23) analysis was performed to determine operational taxonomic units explaining the differences between each group based on a *P* < 0.05 and LDA score > 3.5 and graphed using JMP 16.0.

## Results

### Calves

Of the 160 calves available, 60 calves were randomly chosen for this study based on the enrollment criteria. These 60 calves consisted of 2 groups: 20 of them were in the healthy group, 40 calves with diarrhea. We then removed the calves that received any antimicrobial treatment, which resulted in the diarrheic group to have nine calves removed. Within the healthy calves, 16 had a fecal score of 0, 4 of them had a fecal score of 1, and no calves had a fecal score of 2 or 3. In the diarrheic group, no calves had fecal score of 0 or 1, 8 had a fecal score of 2, and 23 had a fecal score of 3. On the day of arrival, the mean weight differed significantly between healthy calves (109.7 ± 7.6 lbs) and diarrheic calves (103.7 ± 7.74 lbs) (*P* < 0.01). On day 7 (sampling day), the mean weight of healthy calves (113.7 ± 9.5 lbs) was also higher than diarrheic calves (108.6 ± 11.2 lbs) (*P* = 0.09).

### Blood Gas and Electrolytes Measurement

**Figure 1:**
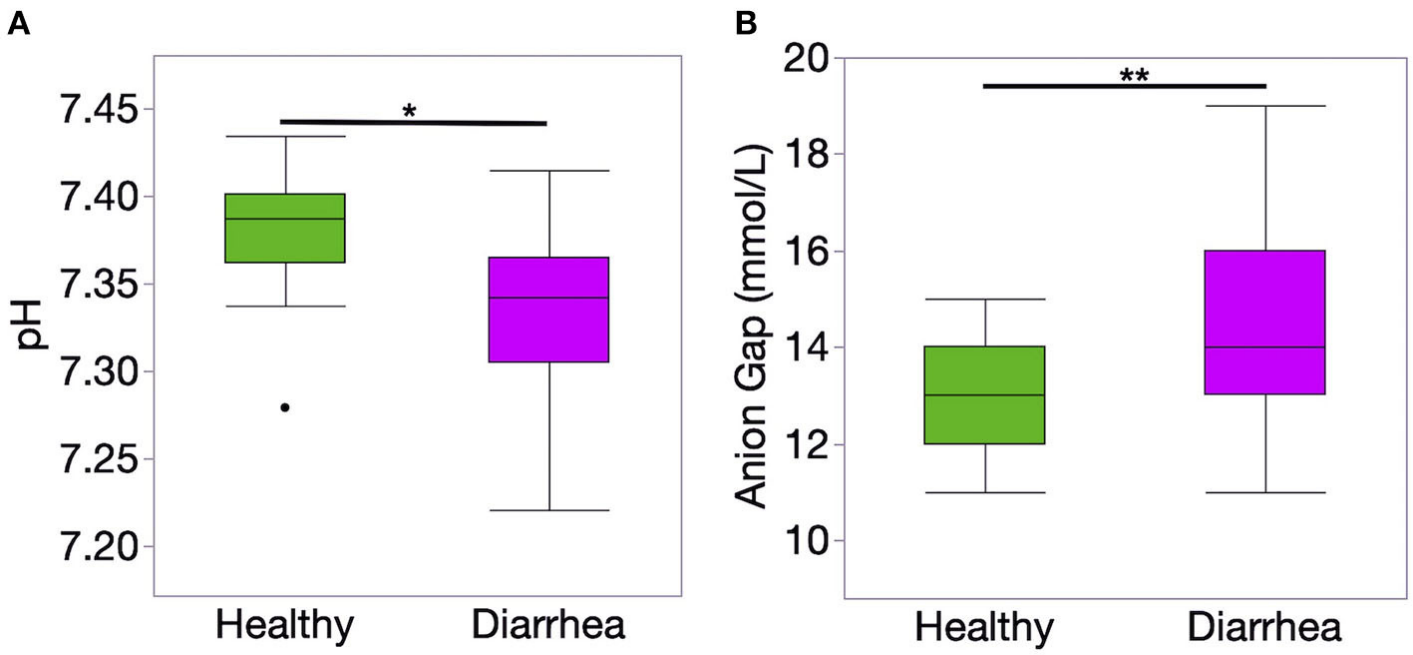
Venous blood ph **(A)** and anion gap (AG) concentration **(B)** of healthy (*n* = 20) and diarrheic (*n* = 31) calves. The AG was calculated using the following formula: AG = (Na + K) – (Cl + HCO${}_{3}^{-}$). Level of significance *P < 0.001 and **P < 0.01. *P*-values were obtained using a *t*-student test.

The results of blood gas and electrolyte analysis is presented in Table 1. Diarrheic calves had lower pH and bicarbonate concentration than healthy calves (*P* < 0.01, for all comparisons). The concentration of unmeasured anions estimated using the AG was higher in diarrheic than healthy calves (*P* = 0.006) (Figure 1).

**Table 1:** Blood gas and electrolyte analysis of healthy and diarrheic calves.

**Variable**	**Healthy**	**Diarrheic**	***P*-value**
	***n* = 20**	***n* = 31**	
PCV (%)	26.4 ± 4.89	29.45 ± 5.10	0.038
Hb (g/dL)	8.98 ± 1.67	10.02 ± 1.73	0.038
pH	7.38 ± 0.03	7.33 ± 0.05	< 0.001
PvCO_2_ (mmHg)	54.9 ± 4.33	55.31 ± 5.98	0.776
HCO_3_ (mmol/L)	32.48 ± 2.08	29.45 ± 5.35	0.006
BE (mmol/L)	7.3 ± 2.30	3.55 ± 6.02	0.003
Anion Gap (mmol/L)	12.95 ± 1.36	14.32 ± 2.09	0.006
Na^+^ (mmol/L)	135.7 ± 1.81	136.81 ± 3.63	0.155
K^+^ (mmol/L)	4.80 ± 0.28	5.05 ± 0.61	0.046
Cl^−^ (mmol/L)	93.55 ± 7.07	98.10 ± 6.08	0.023
Glu (mg/dL)	103.8 ± 22.22	91.13 ± 17.61	0.038
BUN (mmol/L)	9.8 ± 2.46	14.39 ± 9.27	0.012

*PCV, packet cell volume; Hb, Hemoglobin; Venous pressure of carbon dioxide; HCO_3_, Bicarbonate; BE, Base excess; Glu, Glucose; BUN, Blood urea nitrogen. P-values were obtained using a t-student test*.

### Microbiota Sequence Analysis

A total of 10,455,063 raw sequences were obtained, and after filtering and curating 7,739,843 sequences were available for analysis. Subsampling was performed at 92,000 sequences per sample and coverage was adequate based on a Good's coverage index value of 99.9%. In total, 23 phyla, 43 classes, 81 orders, 151 families, and 392 genera were identified in the samples.

### Alpha and Beta Diversity Measurements

**Figure 2:**
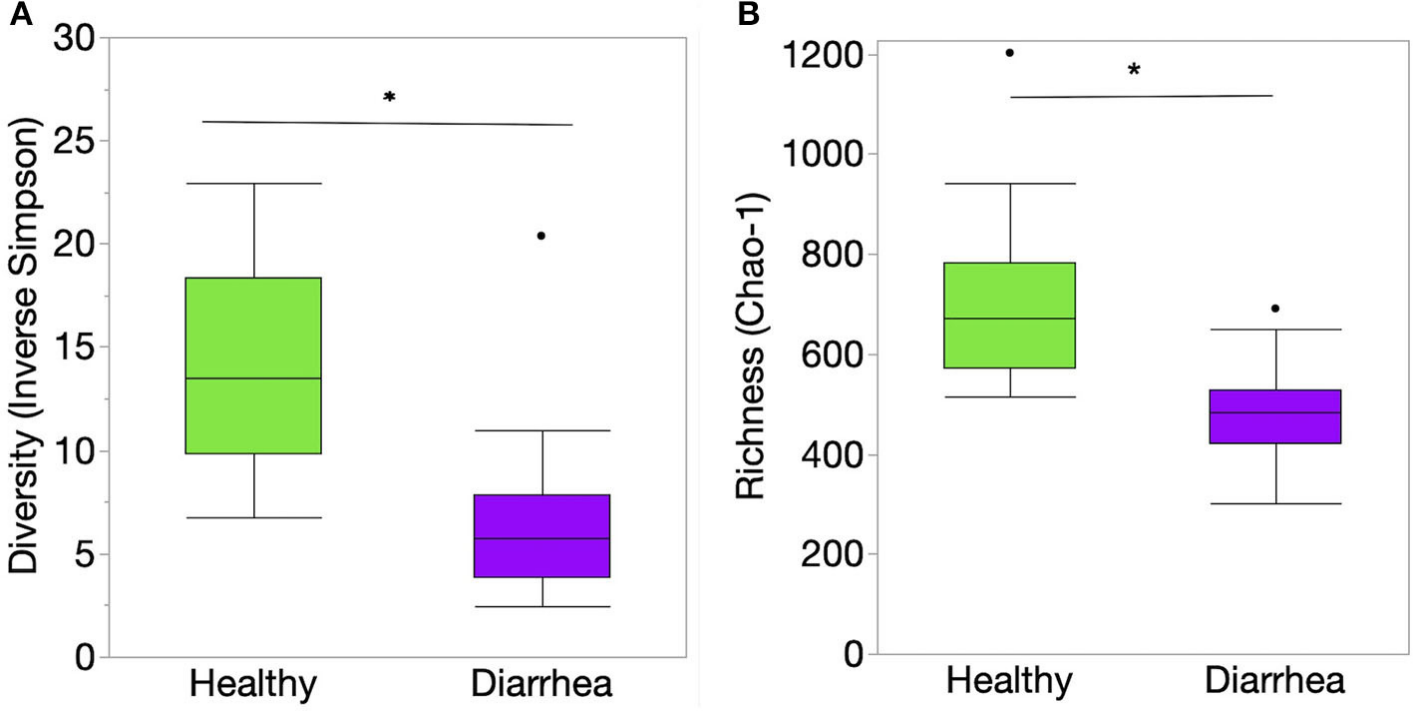
Indices of alpha diversity at the genus level of taxonomy of the fecal samples from healthy and diarrheic calves represented by box and whisker plots. **(A)** Diversity (Inverse Simpson's diversity index); **(B)** Richness (Chao-1 index). Level of significance *P < 0.001. The center line denotes the median value (50th percentile), while the upper and lower bounds of each box represent the 25th and 75th percentiles, respectively. The whiskers mark the 95th and 5th percentiles. Outliers are denoted with dots (·). The *P* values were obtained using the non-parametric Steel-Dwass test for multiple comparisons.

**Figure 3:**
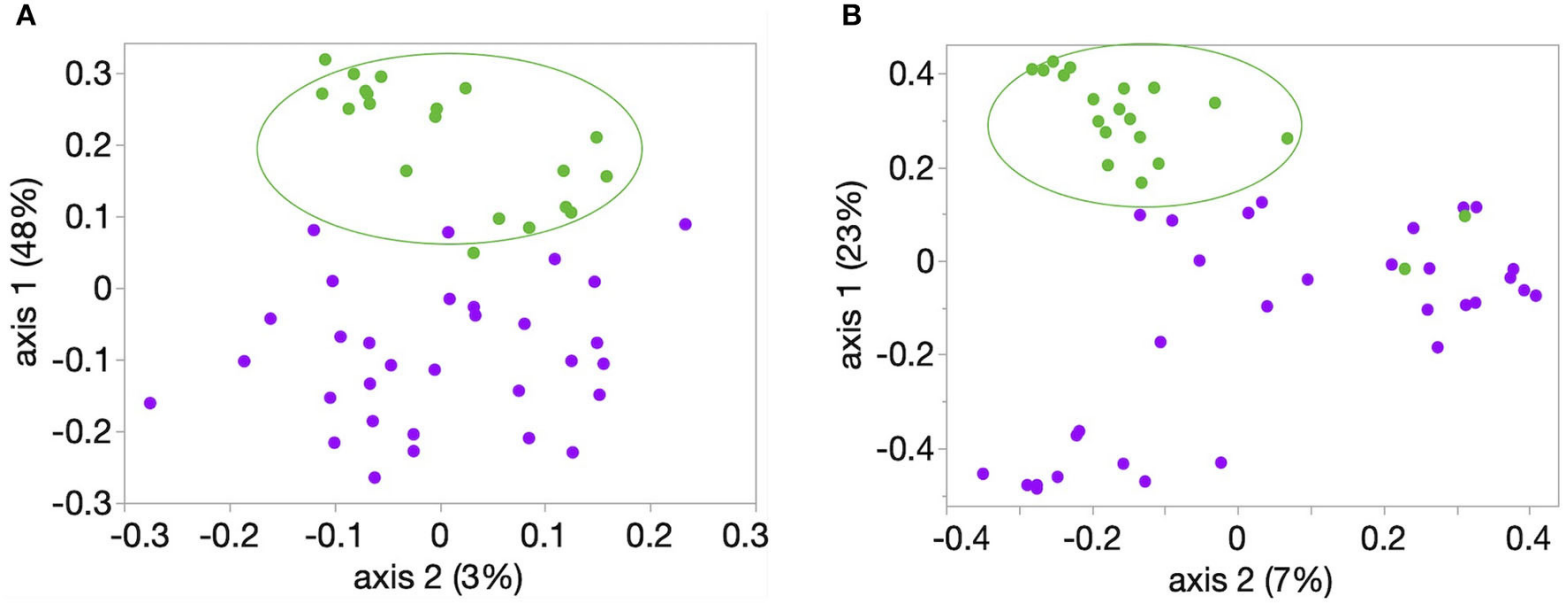
Principal coordinate analysis (PCoA) of bacterial community **(A)** membership (Classic Jaccard analysis) and **(B)** structure (Yue and Clayton analysis). Samples from these 20 healthy (green circles) and 31 calves with diarrhea were analyzed. Comparison between healthy and diarrheic calves showed significant difference in community membership and structure (AMOVA *P* < 0.001, for both comparisons).

Richness, evenness, and diversity were significantly greater in healthy calves compared to diarrheic calves (*P* < 0.001) (Figure 2). The community membership (Jaccard index) and structure (Yu and Clayton index) were different between healthy and diarrheic calves (AMOVA *P* < 0.001) (Figure 3). This was evident by the different clustering of samples from healthy and diarrheic groups (Figure 3).

### Relative Abundance and LefSe Analysis

**Figure 4:**
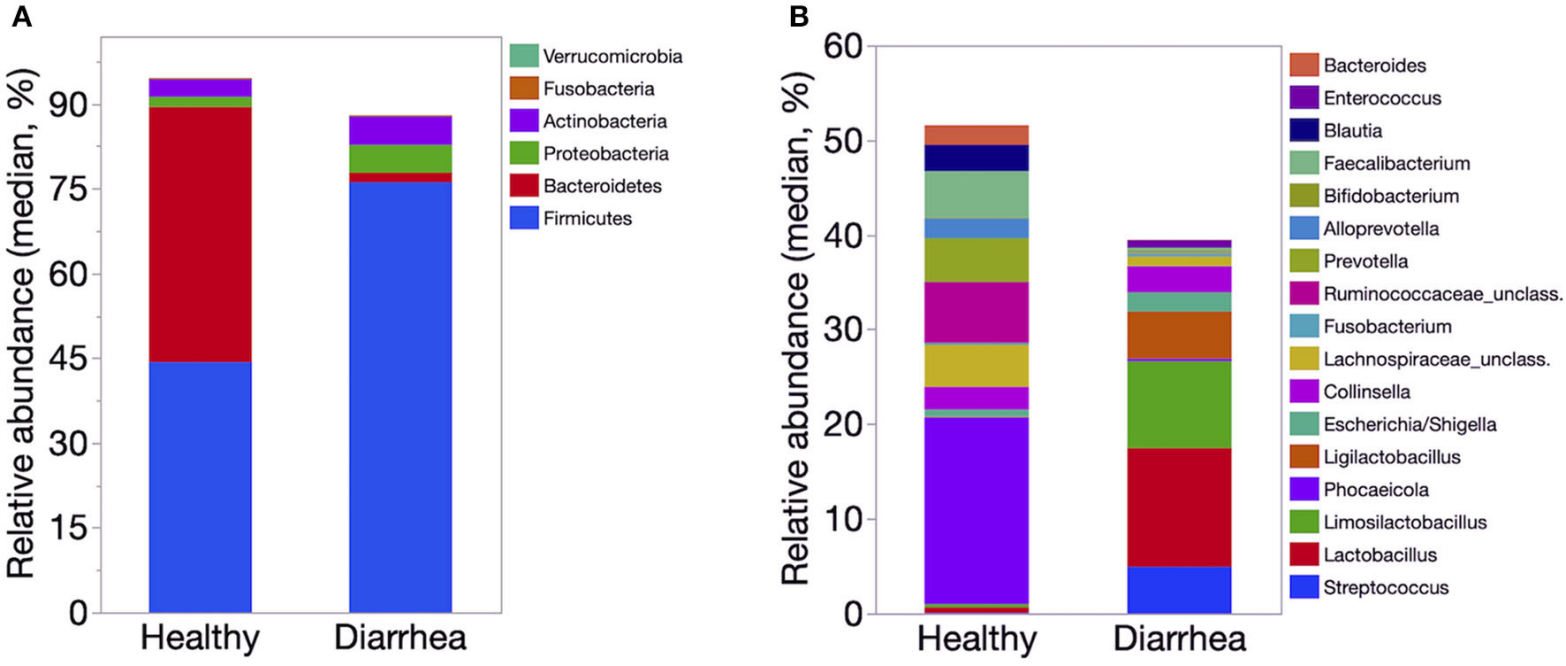
Median relative abundance of predominant bacteria at the phylum **(A)** and genus **(B)** level identified in feces of healthy (*n* = 20) and diarrheic dairy calves (*n* = 31). The 6 most abundant phyla and 17 most abundant genera are displayed.

The relative abundance of Firmicutes was significantly lower in healthy calves than that of diarrheic calves (*P* < 0.01). The abundance of Proteobacteria was significantly higher in the diarrheic group than in the healthy group (*P* = 0.002). The relative abundance of Bacilli and Lactobacillaceae was significantly lower in healthy calves than diarrheic calves (*P* < 0.001). Bacteroidales, *Prevotella, Faecalibacterium*, and *Blautia* were significantly higher in healthy than diarrheic calves (*P* < 0.001). The relative abundance of *Streptococcus* and *Ligilactobacillus* were significantly higher in diarrheic calves compared to healthy calves (*P* < 0.001) (Figure 4).

The LefSe analysis revealed that *Phocaeicola, Bacteroides, Prevotella, Faecalibacterium, Butyricicoccus*, Ruminococcaceae and Lachnospiraceae were enriched in healthy compared to diarrheic calves. The samples from diarrheic calves were enriched with taxa from the phylum Firmicutes, Proteobacteria and Actinobacteria. *Enterococcus, Ligilactobacillus, Lactobacilus*, and *Gallibacterium Streptococcus* were enriched in diarrheic calves (Table 2).

**Table 2:** Taxa enriched in healthy and diarrheic calves identified using the linear discriminatory analysis (LDA) effect size (LefSe) methodology.

**Group**	**Phylum**	**Family**	**Genus**
Healthy *n* = 20	Bacteroidetes	Bacteroidaceae	*Phocaeicola*
			*Bacteroides*
		Prevotellaceae	*Prevotella*
		Odoribacteraceae	*Odoribacter*
		^*^Bacteroidales_unclassified	*unclassified*
	Firmicutes	Ruminococcaceae	*Faecalibacterium*
			*Butyricicoccus*
			*unclassified*
		Lachnospiraceae	*Blautia*
			*unclassified*
		Acidaminococcaceae	*Phascolarctobacterium*
			
Diarrheic *n* =31	Firmicutes	Lactobacillaceae	*Lactobacillus*
			*Ligilactobacillus*
			*Limosilactobacillus*
		Lachnospiraceae	*Mediterraneibacter*
			*unclassified*
		Enterococcaceae	*Enterococcus*
		Pasteurellaceae	*Gallibacterium*
		Streptococcaceae	*Streptococcus*
		Veillonellaceae	*Veillonella*
		Clostridiaceae_1	*Clostridium_sensu_stricto*
	Proteobacteria	Enterobacteriaceae	*Escherichia/Shigella*
	Actinobacteria	Atopobiaceae	*Olsenella*

*LDA cut-off of 3 and P < 0.05*.

* *Taxa classified as Bacteroidales order*.

### Meta-Communities

Using DMM, samples were classified into two different Meta-communities. Meta-community 1 comprised 49% (20/41) of healthy calves, 51% (21/41) diarrheic calves. Meta-community 2 consisted of 100% (10/10) of samples from diarrheic calves.

## Discussion

This study investigated the fecal bacterial alterations of calves with diarrhea and examined the association of those bacterial changes with changes in blood pH, HCO${}_{3}^{-}$ concentration and AG. In diarrheic calves, richness, evenness and diversity were reduced compared to healthy calves, and both community structure and membership were different between healthy and diarrheic calves. These alterations indicated that dysbiosis occurred in the diarrheic calves included in our study. In our study, a shift from obligate anaerobes to facultative anaerobes was characterized by a decrease in Bacteroidales, *Bacteroides, Phocaeicola, Prevotella, Odoribacter* Lachnospiraceae, Ruminococcaeceae, *Butyricicoccus, Blautia* and *Faecalibacterium* and an enrichment of *Streptococcus, Enterococcus, Gallibacterium, and Escherichia/Shigella* in diarrheic calves. This shift from obligate anaerobes to facultative anaerobes have been regarded as a hallmark of dysbiosis of gastrointestinal microbiota in different species (3, 11, 24–27). The gastrointestinal tract is characterized by a low oxygen concentration harboring many obligate anaerobes (10^7^ to 10^11^ g^−1^ colonies), however during dysbiosis in diarrheic calves it is possible that a reduction or disappearance of oxygen-sensitive bacteria (e.g., *Faecalibacterium*) occurs (7, 11, 27). The reduction of taxa (e.g., *Blautia*) that maintain anaerobic conditions in the GIT could have led to a higher oxygen availability in the intestine favoring the proliferation of facultative anaerobes (e.g., *Streptococcus, Enterococcus* Gallibacterium, *and Escherichia/Shigella*) (28). Thus, in diarrheic calves, the reduction in Lachnospiraceae, Ruminococcaeceae, *Butyricicoccus, Blautia* and *Faecalibacterium* could have contributed to the shift from obligate anaerobes to facultative anaerobes. Proliferation of facultative anaerobes in animal models is associated with a proinflammatory mucosal immune response and plays a causative role in gastrointestinal inflammation (3–5, 7, 11, 26–28). During gastrointestinal inflammation, an increased release of hemoglobin carrying oxygen and reactive oxygen species into the lumen of the gastrointestinal tract appear to occur, which could favor the growth of facultative anaerobes (11). Low oxygen tension conditions facilitate proliferation of Enterobacteriaceae and other potentially pathogenic facultative anaerobes (e.g., *Enterococcus, Pseudomonas*), which can elicit an inflammatory response (28). Changes in the luminal pH of the GIT could have also contributed to the bacterial alteration observed in the diarrheic calves, because pH causes selective pressure on bacterial growth and metabolism (29). In horses with grain overload and cattle with ruminal acidosis the increase in facultative anaerobes, especially Enterobacteriaceae along with an acidic luminal environment is associated with cecal and ruminal mucosal damage and facilitate translocation of bacteria and their products into systemic circulation (30–34). This is of interest because bacteremia (35–37) and endotoxemia (38) has been reported in diarrheic calves with bacteremia occurring in 10 to 30% of diarrheic calves with *E. coli* and other facultative anaerobic Enterobacteriaceae bacteria (e.g., *Salmonella, Enterobacter, Klebsiella*) being the most common bacteria isolated (35–37). These findings highlight the crucial role of gastrointestinal bacterial communities in local and systemic inflammatory responses observed in diarrheic calves.

Diarrheic calves had a lower blood pH and bicarbonate concentrations and higher AG values than healthy calves. Increased concentrations of L- and especially D-lactate in the lumen of the GIT and subsequent absorption into systemic circulation play an important role in the local and systemic abnormalities observed in calves with diarrhea (13, 39–41). In horses with carbohydrate overload (30, 31), cattle with ruminal acidosis (42, 43) and humans with SBD (42, 43), luminal concentrations of D- and L-lactate are increased. These alteration are associated with decrease lactic acid fermentation from both proliferation of lactate-producing bacteria and inhibition of lactate fermenting bacteria because of the availability of carbohydrates (30, 34). In our study, lactate-producing bacteria including *Lactobacillu*s, *Streptococcus, Veillonella, Ligilactobacillus* and *Olsenella* were enriched in diarrheic calves. Therefore, the lower blood pH and bicarbonate concentration and the high AG values identified in diarrheic compared to healthy calves was likely due to increased absorption of D- and L-lactate from the lumen of the gastrointestinal tract. The main source of energy and carbohydrates in cow milk is lactose, which is hydrolyzed by lactase in the small intestine to glucose and galactose (44). Lactase is produced and secreted at the brush border of the small intestine (45). Most pathogens associated with calf diarrhea destroy the brush border of the small intestine leading to a decreased activity of lactase (2, 46). In healthy calves under normal lactase activity, only a small percent of ingested lactose will reach the colon (47, 48). When intestinal lactase is low (e.g., during diarrhea) lactose is not metabolized in the small intestine, thus a large amount of lactose enters in the colon (45, 47, 48). Lactate-producing bacteria, such as *Lactobacillu*s, possess β -galactosidase activity and hydrolyze lactose to glucose and galactose (49, 50). Following this hydrolyzation reaction, galactose is then fermented into products such as short chain fatty acids, and D- and L-lactate (50). In diarrheic calves, impaired lactose metabolism has been demonstrated (13, 48). Therefore, the increased luminal concentrations of D- and L-lactate probably resulted from an increased passage of lactose to the distal small intestine and colon due to maldigestion and malabsorption of milk (the only source of food for pre-weaned calves) in the gastrointestinal tract, which may have caused an overgrowth of lactate-producing bacteria (13).

In our study, a single time point fecal sample at the onset of diarrhea was assessed for microbiota analysis, therefore we could not determine whether the differences in fecal microbiota between healthy and diarrheic were already pre-existing and indeed contributed to the susceptibility of the calves to enteropathogens. This is of importance because different farm environmental conditions and management practices such as colostrum administration (51–53), diet (54) or antimicrobial usage at the farm level (55, 56) contribute to the development of the immune system and the colonization and establishment of the bacterial communities in the GIT microbiota (57, 58). Therefore, a longitudinal study involving a large number of farms with multiple management practices is needed to confirm whether the shift from obligated to facultative anaerobes results from the gastrointestinal inflammation or it is a predisposing factor for diarrhea associated with certain management farm practices.

There are some limitations that should be considered when interpreting the results of our study. First, calves of different ages (i.e., 3–7 days) were included in the study. This is of importance because the fecal microbiota of newborn calves changes rapidly during the first days of live (0 to 7 days). However, during sampling, the calf's ages ranged from 10 to 14 days. At that age, the changes of the fecal microbiota are less dramatic than those observed during the first week of life (59–62). Finally, the load of unmeasured anions was estimated using the anion gap which could be affected by changes in blood concentration of total plasma proteins (i.e., albumin and globulin) and phosphates (12) of calves, especially in markedly dehydrated diarrheic calves. Therefore, studies determining the individual concentration of D- and L-lactate in blood and feces of diarrheic calves and their relationship with alterations in the microbiota are warranted. Nonetheless, the results of this study indicate that calf diarrhea is associated with a shift from obligated to facultative anaerobes and expansion of lactate-producing bacteria which are related to acidemia and increased concentrations of unmeasured anions (L- and D-lactate) estimated using the AG.

## Data Availability Statement

The original contributions presented in the study are publicly available. This data can be found here: https://www.ncbi.nlm.nih.gov/sra, BioProject ID: PRJNA795094.

## Ethics Statement

The animal study was reviewed and approved by Animal Care Committee, University of Guelph (Animal Use Protocol no. #4238).

## Author Contributions

DG and DR contributed to the conception and design of the study. DG, LL, HG, LG, and DR contributed to drafting and critically revising the manuscript for important intellectual content. LL and HG contributed to acquisition of data. DG and LL performed the analysis of data. All authors approved the final version to be submitted.

## Funding

Funding for this study was provided by Veal Farmers of Ontario (Guelph, ON, Canada).

## Conflict of Interest

The authors declare that the research was conducted in the absence of any commercial or financial relationships that could be construed as a potential conflict of interest.

## Publisher's Note

All claims expressed in this article are solely those of the authors and do not necessarily represent those of their affiliated organizations, or those of the publisher, the editors and the reviewers. Any product that may be evaluated in this article, or claim that may be made by its manufacturer, is not guaranteed or endorsed by the publisher.
